# Ethyl 3-nitro-4-(propyl­amino)benzoate

**DOI:** 10.1107/S1600536808021314

**Published:** 2008-07-16

**Authors:** Siti Marina Mohd. Maidin, Aisyah Saad Abdul Rahim, Shafida Abdul Hamid, Reza Kia, Hoong-Kun Fun

**Affiliations:** a School of Pharmaceutical Sciences, Universiti Sains Malaysia, 11800 USM, Penang, Malaysia; b School of Chemical Sciences, Universiti Sains Malaysia, 11800 USM, Penang, Malaysia; c X-ray Crystallography Unit, School of Physics, Universiti Sains Malaysia, 11800 USM, Penang, Malaysia

## Abstract

In the title compound, C_12_H_16_N_2_O_4_, intra­molecular N—H⋯O and C—H⋯O hydrogen bonds generate *S*(6) and *S*(5) ring motifs, respectively. The nitro group is almost coplanar with the benzene ring, forming a dihedral angle of 6.2 (2)°. In the crystal structure, neighbouring mol­ecules are linked together by inter­molecular N—H⋯O and O⋯O inter­actions. Of interest are the short inter­molecular O⋯O inter­actions which cause a stacking arrangement of the mol­ecules along the *a* axis.

## Related literature

For related literature on hydrogen-bond motifs, see: Bernstein *et al.* (1995 ▶). For bond-length data, see: Allen *et al.* (1987 ▶). For related literature, see: Ishida *et al.* (2006 ▶); Vinodkumar *et al.* (2008 ▶). Rida *et al.* (2005 ▶); Harikrishnan *et al.* (2008 ▶); Moore *et al.* (2005 ▶).
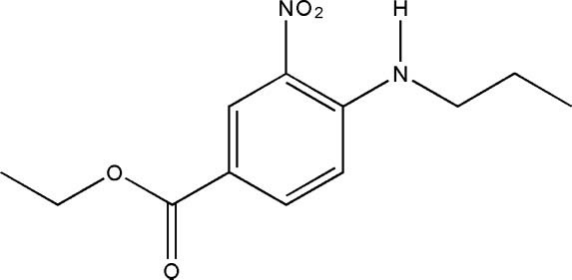


## Experimental

### 

#### Crystal data

C_12_H_16_N_2_O_4_
*M* *_r_* = 252.27
Triclinic, 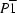

*a* = 4.4914 (4) Å
*b* = 12.0828 (9) Å
*c* = 12.8763 (9) Å
α = 62.494 (4)°
β = 81.055 (4)°
γ = 83.494 (4)°
*V* = 611.57 (8) Å^3^
*Z* = 2
Mo *K*α radiation
μ = 0.10 mm^−1^
*T* = 100.0 (1) K
0.51 × 0.26 × 0.26 mm

#### Data collection

Bruker SMART APEXII CCD area-detector diffractometer
Absorption correction: multi-scan (*SADABS*; Bruker, 2005 ▶) *T*_min_ = 0.949, *T*_max_ = 0.974
12286 measured reflections
2372 independent reflections
1946 reflections with *I* > 2σ(*I*)
*R*_int_ = 0.041

#### Refinement

*R*[*F* ^2^ > 2σ(*F* ^2^)] = 0.076
*wR*(*F* ^2^) = 0.252
*S* = 1.17
2372 reflections
169 parameters
H atoms treated by a mixture of independent and constrained refinement
Δρ_max_ = 0.47 e Å^−3^
Δρ_min_ = −0.33 e Å^−3^

### 

Data collection: *APEX2* (Bruker, 2005 ▶); cell refinement: *APEX2*; data reduction: *SAINT* (Bruker, 2005 ▶); program(s) used to solve structure: *SHELXTL* (Sheldrick, 2008 ▶); program(s) used to refine structure: *SHELXTL*; molecular graphics: *SHELXTL*; software used to prepare material for publication: *SHELXTL* and *PLATON* (Spek, 2003 ▶).

## Supplementary Material

Crystal structure: contains datablocks global, I. DOI: 10.1107/S1600536808021314/at2590sup1.cif

Structure factors: contains datablocks I. DOI: 10.1107/S1600536808021314/at2590Isup2.hkl

Additional supplementary materials:  crystallographic information; 3D view; checkCIF report

## Figures and Tables

**Table 1:** Selected interatomic distances (Å)

O1⋯O1^i^	2.914 (5)
O1⋯O1^ii^	2.984 (5)

Symmetry codes: (i) 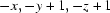; (ii) 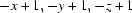.

**Table 2:** Hydrogen-bond geometry (Å, °)

*D*—H⋯*A*	*D*—H	H⋯*A*	*D*⋯*A*	*D*—H⋯*A*
N1—H1*N*1⋯O1	0.85 (5)	2.00 (6)	2.633 (5)	131 (5)
N1—H1*N*1⋯O1^i^	0.85 (5)	2.28 (5)	2.998 (4)	141 (5)
C2—H2*A*⋯O2	0.93	2.35	2.674 (4)	100

Symmetry code: (i) 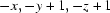.

**Fig. 1.**
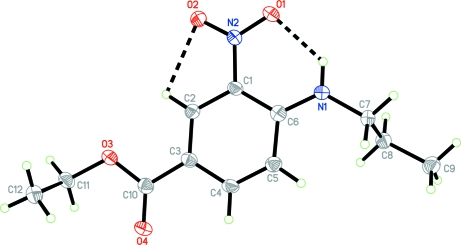
The molecular structure of the title compound, showing 50% probability displacement ellipsoids and the atomic numbering. Intramolecular hydrogen bonds are drawn as dashed lines.

**Fig. 2.**
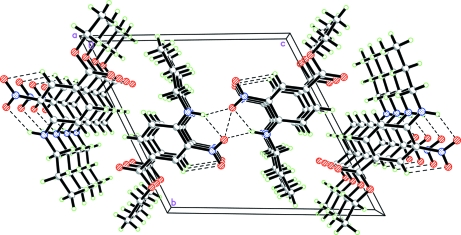
The crystal packing of (I), showing stacking arrangement viewed down the a-axis. Intramolecular and intermolecular interactions are drawn as dashed lines.

## supplementary crystallographic information

### Comment

The benzoic acid cores are precursors to many medicinally important heterocycles, *e.g.* benzimidazoles (Ishida *et al.*, 2006; Vinodkumar *et al.*, 2008) and benzoxazoles (Rida *et al.*, 2005; Harikrishnan *et al.*, 2008). Using Moore's procedure (Moore *et al.*, 2005) with some modifications, we attempted the derivatization of nitro benzoic acid precursors, which led to the synthesis of the title compound (I) bearing a propylamine motif. Its crystal structure has been determined and is presented here.

In the title compound (I), (Fig. 1), intramolecular N—H···O and C—H···O hydrogen bonds generate *S*(6) and *S*(5) ring motifs, respectively (Bernstein *et al.*, 1995). The bond lengths and angles are within normal ranges (Allen *et al.*, 1987).The nitro group is almost coplanar with the benzene ring with torsion angle of -6.4 (5)°. In the crystal structure (Fig. 2), neighbouring molecules are linked together by intermolecular N—H···O and O···O interactions. The interesting feature of the crystal structure is the short intermolecular O···O [symmetry codes: -*x*, 1 - *y*, 1 - *z*; 1 - *x*, 1 - *y*, 1 - *z*] interactions (Table 2) with distances of 2.914 (5) and 2.984 (5) Å which are shorter than the sum of the van der Waals radii of oxygen atoms. These interactions along with the intermolecular N—H···O interactions stack the molecules along the *a* axis.

### Experimental

The title compound (I) was synthesized by adding *N,N*-diisopropyl ethylamine (DIPEA) (0.20 ml, 1.12 mmol) dropwise to a stirred solution of ethyl 4-fluoro-3-nitrobenzoate (200 mg, 0.93 mmol) in dry dichloromethane (10 ml). Propylamine (0.10 ml, 1.03 mmol) was added slowly with stirring, and then the mixture was stirred overnight at room temperature under N_2_. After completion of the reaction, the mixture was washed with 10% Na_2_CO_3_ (10 ml). The aqueous layer was washed again with dichloromethane (3 *x* 15 ml). The organic fractions were pooled and dried over MgSO_4_ and the solvent was removed by rotary evaporator. Recrystallization with hot hexane afforded the desired compound (I) as yellow needle-like crystals.

### Refinement

The H-atom bound to N1 was located from the difference Fourier map and refined freely. The rest of the hydrogen atoms were positioned geometrically [C—H = 0.93–0.97 Å] and refined using a riding model with *U*_iso_ = 1.2 or 1.5*U*_eq_(C). A rotating-group model was used for the methyl groups.

### Crystal data

**Table:**

C_12_H_16_N_2_O_4_	*Z* = 2
*M**_r_* = 252.27	*F*_000_ = 268
Triclinic, *P*1	*D*_x_ = 1.370 Mg m^−^^3^
Hall symbol: -P 1	Mo *K*α radiation λ = 0.71073 Å
*a* = 4.4914 (4) Å	Cell parameters from 6896 reflections
*b* = 12.0828 (9) Å	θ = 3.2–33.0º
*c* = 12.8763 (9) Å	µ = 0.10 mm^−^^1^
α = 62.494 (4)º	*T* = 100.0 (1) K
β = 81.055 (4)º	Needle, yellow
γ = 83.494 (4)º	0.51 × 0.26 × 0.26 mm
*V* = 611.57 (8) Å^3^	

### Data collection

**Table:**

Bruker SMART APEXII CCD area-detector diffractometer	2372 independent reflections
Radiation source: fine-focus sealed tube	1946 reflections with *I* > 2σ(*I*)
Monochromator: graphite	*R*_int_ = 0.041
*T* = 100.0(1) K	θ_max_ = 26.0º
φ and ω scans	θ_min_ = 1.8º
Absorption correction: multi-scan(SADABS; Bruker, 2005)	*h* = −5→5
*T*_min_ = 0.949, *T*_max_ = 0.974	*k* = −14→14
12286 measured reflections	*l* = −15→15

### Refinement

**Table:**

Refinement on *F*^2^	Secondary atom site location: difference Fourier map
Least-squares matrix: full	Hydrogen site location: inferred from neighbouring sites
*R*[*F*^2^ > 2σ(*F*^2^)] = 0.076	H atoms treated by a mixture of independent and constrained refinement
*wR*(*F*^2^) = 0.252	*w* = 1/[σ^2^(*F*_o_^2^) + (0.1098*P*)^2^ + 1.609*P*] where *P* = (*F*_o_^2^ + 2*F*_c_^2^)/3
*S* = 1.17	(Δ/σ)_max_ < 0.001
2372 reflections	Δρ_max_ = 0.47 e Å^−^^3^
169 parameters	Δρ_min_ = −0.33 e Å^−^^3^
Primary atom site location: structure-invariant direct methods	Extinction correction: none

### Special details

**Table:**

Experimental. The low-temperature data was collected with the Oxford Cyrosystem Cobra low-temperature attachment.
Geometry. All e.s.d.'s (except the e.s.d. in the dihedral angle between two l.s. planes) are estimated using the full covariance matrix. The cell e.s.d.'s are taken into account individually in the estimation of e.s.d.'s in distances, angles and torsion angles; correlations between e.s.d.'s in cell parameters are only used when they are defined by crystal symmetry. An approximate (isotropic) treatment of cell e.s.d.'s is used for estimating e.s.d.'s involving l.s. planes.
Refinement. Refinement of *F*^2^ against ALL reflections. The weighted *R*-factor *wR* and goodness of fit *S* are based on *F*^2^, conventional *R*-factors *R* are based on *F*, with *F* set to zero for negative *F*^2^. The threshold expression of *F*^2^ > σ(*F*^2^) is used only for calculating *R*-factors(gt) *etc*. and is not relevant to the choice of reflections for refinement. *R*-factors based on *F*^2^ are statistically about twice as large as those based on *F*, and *R*- factors based on ALL data will be even larger.

### Fractional atomic coordinates and isotropic or equivalent isotropic displacement parameters (Å^2^)

**Table:**

	*x*	*y*	*z*	*U*_iso_*/*U*_eq_	
O1	0.2464 (7)	0.4136 (3)	0.5558 (2)	0.0326 (7)	
O2	0.5971 (6)	0.2725 (2)	0.6301 (2)	0.0316 (7)	
O3	0.8766 (6)	0.1455 (2)	1.0099 (2)	0.0239 (6)	
O4	0.7178 (6)	0.2578 (2)	1.1092 (2)	0.0272 (6)	
N1	0.0274 (7)	0.5600 (3)	0.6530 (3)	0.0217 (7)	
N2	0.4117 (7)	0.3535 (3)	0.6346 (3)	0.0228 (7)	
C1	0.3823 (8)	0.3799 (3)	0.7345 (3)	0.0195 (7)	
C2	0.5528 (8)	0.3013 (3)	0.8258 (3)	0.0200 (7)	
H2A	0.6775	0.2374	0.8192	0.024*	
C3	0.5379 (8)	0.3177 (3)	0.9255 (3)	0.0200 (7)	
C4	0.3490 (8)	0.4159 (3)	0.9327 (3)	0.0210 (7)	
H4A	0.3358	0.4274	0.9999	0.025*	
C5	0.1849 (8)	0.4945 (3)	0.8436 (3)	0.0215 (7)	
H5A	0.0650	0.5589	0.8512	0.026*	
C6	0.1917 (8)	0.4809 (3)	0.7389 (3)	0.0203 (7)	
C7	−0.1636 (8)	0.6655 (3)	0.6552 (3)	0.0223 (7)	
H7A	−0.2755	0.6388	0.7330	0.027*	
H7B	−0.3086	0.6895	0.5991	0.027*	
C8	0.0108 (8)	0.7792 (3)	0.6262 (3)	0.0238 (8)	
H8A	0.1518	0.7572	0.6834	0.029*	
H8B	0.1251	0.8065	0.5488	0.029*	
C9	−0.2061 (9)	0.8850 (3)	0.6280 (3)	0.0281 (8)	
H9A	−0.0932	0.9532	0.6169	0.042*	
H9B	−0.3304	0.8557	0.7025	0.042*	
H9C	−0.3313	0.9126	0.5657	0.042*	
C10	0.7166 (8)	0.2397 (3)	1.0240 (3)	0.0212 (7)	
C11	1.0559 (8)	0.0633 (3)	1.1042 (3)	0.0253 (8)	
H11A	1.1345	0.1115	1.1358	0.030*	
H11B	1.2257	0.0261	1.0728	0.030*	
C12	0.8678 (9)	−0.0386 (3)	1.2015 (3)	0.0292 (8)	
H12A	0.9926	−0.0943	1.2607	0.044*	
H12B	0.7837	−0.0843	1.1696	0.044*	
H12C	0.7077	−0.0020	1.2361	0.044*	
H1N1	0.031 (11)	0.547 (4)	0.593 (5)	0.038 (13)*	

### Atomic displacement parameters (Å^2^)

**Table:**

	*U*^11^	*U*^22^	*U*^33^	*U*^12^	*U*^13^	*U*^23^
O1	0.0432 (16)	0.0348 (15)	0.0271 (14)	0.0142 (12)	−0.0198 (12)	−0.0189 (12)
O2	0.0420 (16)	0.0317 (14)	0.0240 (14)	0.0155 (12)	−0.0099 (11)	−0.0171 (11)
O3	0.0281 (13)	0.0254 (13)	0.0187 (12)	0.0036 (10)	−0.0072 (10)	−0.0099 (10)
O4	0.0372 (15)	0.0284 (13)	0.0185 (12)	0.0009 (11)	−0.0075 (10)	−0.0120 (11)
N1	0.0265 (16)	0.0218 (14)	0.0198 (15)	0.0033 (12)	−0.0064 (12)	−0.0117 (12)
N2	0.0290 (16)	0.0206 (14)	0.0189 (14)	0.0030 (12)	−0.0069 (12)	−0.0087 (12)
C1	0.0248 (17)	0.0185 (16)	0.0145 (16)	−0.0023 (13)	−0.0022 (13)	−0.0065 (13)
C2	0.0232 (17)	0.0182 (15)	0.0186 (16)	−0.0010 (13)	−0.0024 (13)	−0.0084 (13)
C3	0.0211 (17)	0.0211 (16)	0.0170 (16)	−0.0035 (13)	−0.0014 (13)	−0.0077 (13)
C4	0.0275 (18)	0.0212 (16)	0.0143 (15)	−0.0045 (13)	−0.0003 (13)	−0.0080 (13)
C5	0.0228 (17)	0.0216 (16)	0.0201 (17)	−0.0017 (13)	−0.0005 (13)	−0.0100 (14)
C6	0.0200 (16)	0.0200 (16)	0.0199 (17)	−0.0032 (13)	−0.0015 (13)	−0.0080 (13)
C7	0.0235 (17)	0.0215 (17)	0.0219 (17)	0.0052 (13)	−0.0060 (13)	−0.0101 (14)
C8	0.0248 (18)	0.0235 (17)	0.0215 (17)	0.0016 (14)	−0.0030 (13)	−0.0094 (14)
C9	0.033 (2)	0.0240 (18)	0.0261 (19)	0.0008 (15)	−0.0019 (15)	−0.0112 (15)
C10	0.0228 (17)	0.0204 (16)	0.0188 (16)	−0.0036 (13)	−0.0012 (13)	−0.0074 (13)
C11	0.0249 (18)	0.0290 (18)	0.0208 (17)	0.0048 (14)	−0.0080 (14)	−0.0101 (15)
C12	0.034 (2)	0.0262 (18)	0.0247 (19)	0.0051 (15)	−0.0066 (15)	−0.0101 (15)

### Geometric parameters (Å, °)

**Table:**

O1—N2	1.241 (4)	C5—C6	1.426 (5)
O2—N2	1.228 (4)	C5—H5A	0.9300
O3—C10	1.345 (4)	C7—C8	1.524 (5)
O3—C11	1.455 (4)	C7—H7A	0.9700
O4—C10	1.214 (4)	C7—H7B	0.9700
N1—C6	1.342 (4)	C8—C9	1.523 (5)
N1—C7	1.462 (4)	C8—H8A	0.9700
N1—H1N1	0.86 (5)	C8—H8B	0.9700
N2—C1	1.445 (4)	C9—H9A	0.9600
C1—C2	1.398 (5)	C9—H9B	0.9600
C1—C6	1.428 (5)	C9—H9C	0.9600
C2—C3	1.378 (5)	C11—C12	1.511 (5)
C2—H2A	0.9300	C11—H11A	0.9700
C3—C4	1.409 (5)	C11—H11B	0.9700
C3—C10	1.477 (5)	C12—H12A	0.9600
C4—C5	1.364 (5)	C12—H12B	0.9600
C4—H4A	0.9300	C12—H12C	0.9600
			
O1···O1^i^	2.914 (5)	O1···O1^ii^	2.984 (5)
			
C10—O3—C11	116.1 (3)	C8—C7—H7B	108.8
C6—N1—C7	125.0 (3)	H7A—C7—H7B	107.7
C6—N1—H1N1	118 (3)	C9—C8—C7	110.2 (3)
C7—N1—H1N1	117 (3)	C9—C8—H8A	109.6
O2—N2—O1	121.8 (3)	C7—C8—H8A	109.6
O2—N2—C1	119.5 (3)	C9—C8—H8B	109.6
O1—N2—C1	118.6 (3)	C7—C8—H8B	109.6
C2—C1—C6	122.1 (3)	H8A—C8—H8B	108.1
C2—C1—N2	116.1 (3)	C8—C9—H9A	109.5
C6—C1—N2	121.8 (3)	C8—C9—H9B	109.5
C3—C2—C1	120.6 (3)	H9A—C9—H9B	109.5
C3—C2—H2A	119.7	C8—C9—H9C	109.5
C1—C2—H2A	119.7	H9A—C9—H9C	109.5
C2—C3—C4	118.4 (3)	H9B—C9—H9C	109.5
C2—C3—C10	123.3 (3)	O4—C10—O3	123.5 (3)
C4—C3—C10	118.2 (3)	O4—C10—C3	123.7 (3)
C5—C4—C3	121.6 (3)	O3—C10—C3	112.8 (3)
C5—C4—H4A	119.2	O3—C11—C12	110.8 (3)
C3—C4—H4A	119.2	O3—C11—H11A	109.5
C4—C5—C6	122.0 (3)	C12—C11—H11A	109.5
C4—C5—H5A	119.0	O3—C11—H11B	109.5
C6—C5—H5A	119.0	C12—C11—H11B	109.5
N1—C6—C5	120.4 (3)	H11A—C11—H11B	108.1
N1—C6—C1	124.4 (3)	C11—C12—H12A	109.5
C5—C6—C1	115.2 (3)	C11—C12—H12B	109.5
N1—C7—C8	113.8 (3)	H12A—C12—H12B	109.5
N1—C7—H7A	108.8	C11—C12—H12C	109.5
C8—C7—H7A	108.8	H12A—C12—H12C	109.5
N1—C7—H7B	108.8	H12B—C12—H12C	109.5
			
O2—N2—C1—C2	−5.7 (5)	C4—C5—C6—C1	−0.1 (5)
O1—N2—C1—C2	174.0 (3)	C2—C1—C6—N1	178.9 (3)
O2—N2—C1—C6	173.9 (3)	N2—C1—C6—N1	−0.5 (5)
O1—N2—C1—C6	−6.5 (5)	C2—C1—C6—C5	−0.9 (5)
C6—C1—C2—C3	1.2 (5)	N2—C1—C6—C5	179.6 (3)
N2—C1—C2—C3	−179.2 (3)	C6—N1—C7—C8	78.2 (4)
C1—C2—C3—C4	−0.5 (5)	N1—C7—C8—C9	178.8 (3)
C1—C2—C3—C10	−178.7 (3)	C11—O3—C10—O4	0.3 (5)
C2—C3—C4—C5	−0.5 (5)	C11—O3—C10—C3	−179.2 (3)
C10—C3—C4—C5	177.8 (3)	C2—C3—C10—O4	175.7 (3)
C3—C4—C5—C6	0.9 (5)	C4—C3—C10—O4	−2.5 (5)
C7—N1—C6—C5	1.2 (5)	C2—C3—C10—O3	−4.8 (5)
C7—N1—C6—C1	−178.6 (3)	C4—C3—C10—O3	177.0 (3)
C4—C5—C6—N1	180.0 (3)	C10—O3—C11—C12	85.5 (4)

Symmetry codes: (i) −*x*, −*y*+1, −*z*+1; (ii) −*x*+1, −*y*+1, −*z*+1.

### Hydrogen-bond geometry (Å, °)

**Table:**

*D*—H···*A*	*D*—H	H···*A*	*D*···*A*	*D*—H···*A*
N1—H1N1···O1	0.85 (5)	2.00 (6)	2.633 (5)	131 (5)
N1—H1N1···O1^i^	0.85 (5)	2.28 (5)	2.998 (4)	141 (5)
C2—H2A···O2	0.93	2.35	2.674 (4)	100

Symmetry codes: (i) −*x*, −*y*+1, −*z*+1.
